# Insight into Population Structure and Drug Resistance of Pediatric Tuberculosis Strains from China and Russia Gained through Whole-Genome Sequencing

**DOI:** 10.3390/ijms241210302

**Published:** 2023-06-18

**Authors:** Svetlana Zhdanova, Wei-Wei Jiao, Viacheslav Sinkov, Polina Khromova, Natalia Solovieva, Alexander Mushkin, Igor Mokrousov, Olesya Belopolskaya, Aleksey Masharsky, Anna Vyazovaya, Lubov Rychkova, Lubov Kolesnikova, Viacheslav Zhuravlev, A-Dong Shen, Oleg Ogarkov

**Affiliations:** 1 Department of Epidemiology and Microbiology, Scientific Centre for Family Health and Human Reproduction Problems, 664003 Irkutsk, Russia; 2 National Clinical Research Center for Respiratory Diseases, Beijing Key Laboratory of Pediatric Respiratory Infection Disease, Beijing Pediatric Research Institute, Beijing Children’s Hospital, Capital Medical University, National Center for Children’s Health, Beijing 100045, China; 3 St. Petersburg Research Institute of Phthisiopulmonology, 191036 St. Petersburg, Russia; 4 Laboratory of Molecular Epidemiology and Evolutionary Genetics, St. Petersburg Pasteur Institute, 197101 St. Petersburg, Russia; 5 Henan International Joint Laboratory of Children’s Infectious Diseases, Children’s Hospital Affiliated to Zhengzhou University, Henan Children’s Hospital Zhengzhou Children’s Hospital, Zhengzhou 450012, China; 6 The Bio-Bank Resource Center, Research Park, St. Petersburg State University, 199034 St. Petersburg, Russia

**Keywords:** *Mycobacterium tuberculosis*, pediatric tuberculosis, drug resistance, Russia, China

## Abstract

This study aimed to determine phenotypic and genotypic drug resistance patterns of *Mycobacterium tuberculosis* strains from children with tuberculosis (TB) in China and Russia, two high-burden countries for multi/extensively-drug resistant (MDR/XDR) TB. Whole-genome sequencing data of *M. tuberculosis* isolates from China (*n* = 137) and Russia (*n* = 60) were analyzed for phylogenetic markers and drug-resistance mutations, followed by comparison with phenotypic susceptibility data. The Beijing genotype was detected in 126 Chinese and 50 Russian isolates. The Euro-American lineage was detected in 10 Russian and 11 Chinese isolates. In the Russian collection, the Beijing genotype and Beijing B0/W148-cluster were dominated by MDR strains (68% and 94%, respectively). Ninety percent of B0/W148 strains were phenotypically pre-XDR. In the Chinese collection, neither of the Beijing sublineages was associated with MDR/pre-XDR status. MDR was mostly caused by low fitness cost mutations (*rpoB* S450L, *katG* S315T, *rpsL* K43R). Chinese rifampicin-resistant strains demonstrated a higher diversity of resistance mutations than Russian isolates (*p* = 0.003). The rifampicin and isoniazid resistance compensatory mutations were detected in some MDR strains, but they were not widespread. The molecular mechanisms of *M. tuberculosis* adaptation to anti-TB treatment are not unique to the pediatric strains, but they reflect the general situation with TB in Russia and China.

## 1. Introduction

Multidrug-resistant tuberculosis (MDR-TB, defined as TB resistant to at least rifampicin and isoniazid), is a major threat to TB control programs. In 2021, about 450,000 cases of MDR-TB were reported [1], which implies a high cost of treatment (€764 EUR is the cost for a shorter regimen, including bedaquiline for six months; €8709 EUR is the cost for pre-extensively drug-resistant tuberculosis per person [2] compared to drug-susceptible TB only; only 43% (649,000) of them received treatment (in 2018 to 2021), and its effectiveness was low [1]. According to the most recent Global Tuberculosis Report 2022, Russia is among the top three countries with the largest share of incident cases of MDR/RR-TB in 2021 with 8.5% of global cases, preceded by India (26% of global cases) and followed by Pakistan (7.9% of global cases) [1]. The prevalence of MDR-TB in Russia suggests that epidemic transmission, primarily from adults to children, plays an important role in pediatric MDR-TB in this country. The TB incidence in children (zero tofourteen years) decreased two-fold from 16.4 in 2006 to 7.7 in 2019, per 100,000 children, due to the overall improvement of TB care and control in Russia, but the proportion of children with MDR-TB increased (2005—10.0%; 2019—45.8%), similar to the situation in the general adult population [3].

In China, the drug resistance survey in 2015 found that the MDR-TB rate was 8.6% and 23.2% in newly-diagnosed and retreatment patients, respectively, in the general population [4]. In Jilin, China, the rate of primary MDR-TB was 8.6% of bacteriologically confirmed cases. Among these primary MDR-TB cases, one-third were associated with recent human-to-human transmission [5,6]. Regarding pediatric TB in China, in the studies carried out in different parts of the country, the drug resistance rate varied from 19% to 31%, and the MDR-TB rate varied from 4.6% to 6.9% in 2003–2018 [7,8,9].

In 2021, the WHO changed the definition of extensively drug-resistant (XDR) TB, and pre-XDR. Pre-XDR TB is MDR-TB with additional resistance to any fluoroquinolone. XDR-TB is MDR-TB resistant to any fluoroquinolone and at least one additional new second-line drug, bedaquiline and/or linezolid [10,11]. The primary mechanism for acquiring resistance in *M. tuberculosis* is the accumulation of mutations in genes associated with resistance and the selection of mutant alleles due to inadequate or interrupted treatment [12]. Drug resistance in mycobacteria was also suggested to be acquired through drug inactivation, activation of efflux pumps, change in membrane permeability, intrinsic bacterial resistance, general persistence and tolerance, and post-translational modifications of the proteins that interact with the drugs (reviewed in [13,14]). Transmission of drug-resistant strains is another important reason for primary resistant TB [15]. The transmission model shows that the epidemic spread of MDR-TB is significantly increasing and varies in different countries from 40% to 90% [15].

Whole genome sequencing (WGS) gives detailed knowledge of the relationship between *M. tuberculosis* strain genotypes, drug resistance properties, and all drug-resistance alleles. WGS allows high-throughput analysis of all mutations to determine the prevailing resistance pattern and to assess their frequency.

China and Russia are countries with a high burden of drug-resistant TB and high diversity of the locally circulating *M. tuberculosis* strains. Both countries have considerable impact on global health and economy, and both feature the worrisome emergence of *M. tuberculosis* strains resistant to the new-generation drugs and their active transmission.

The objective of this study was to comprehensively detect the *M. tuberculosis* genetic diversity underlying anti-TB drug-resistance through WGS analysis of pediatric *M. tuberculosis* strains from China and Russia, two high-burden countries of MDR-TB. To this end, we used the WHO-endorsed catalogue of molecular targets for *M. tuberculosis* drug susceptibility testing (DST) [16]. In addition, we determined the phylogenetically informative genetic variation and analyzed the distribution of drug resistance among lineages and genotype clusters of the studied strains. We compared genomic diversity observed in pediatric strains against available knowledge of adult strains from the same country. Furthermore, *M. tuberculosis* strains from children are known to be particularly difficult to isolate [17,18,19], and, in this sense, we believe that the unique value of this study is that it included sufficiently large collections of pediatric *M. tuberculosis* isolates from Russia and China.

## 2. Results

### 2.1. Phylogeny and Population Structure of Pediatric M. tuberculosis Strains in China and Russia

**Figure 1:**
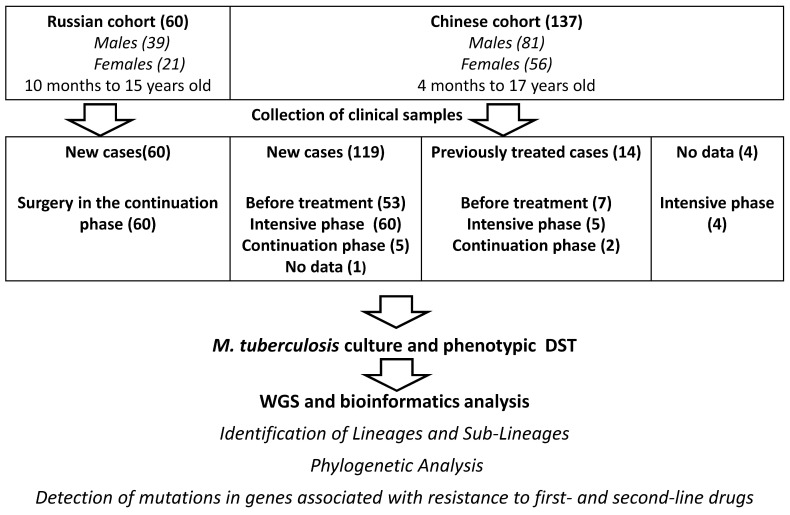
Flowchart of the study of Russian and Chinese pediatric *M. tuberculosis* strains. DST—drug susceptibility testing.

The flowchart of the study design is shown in Figure 1. The demographic information of the 197 children with TB and their strains is shown in Table S1. Russian and Chinese cohorts of patients showed similar ages (four months to seventeen years old for Chinese children and ten months to fifteen years old for Russian children) and sex distributions. Of 197 isolates, 107 isolates were susceptible to all tested drugs, 65 were resistant to both rifampicin (RIF) and isoniazid (INH), and 15 were pre-XDR by phenotypic tests. Five isolates (three Russian and two Chinese) with confirmed MDR and pre-XDR phenotypes, but without known mutations in resistance genes, were considered resistant in this study (see Table S2 and below).

High-quality WGS data were generated for 197 isolates with a median of 5,251,491 read pairs per isolate. Mapping of the reads resulted in an average depth of >100-fold coverage for 97.5% of genome positions, as well as at least 80x coverage for 2.5% of the genome positions.

**Figure 2:**
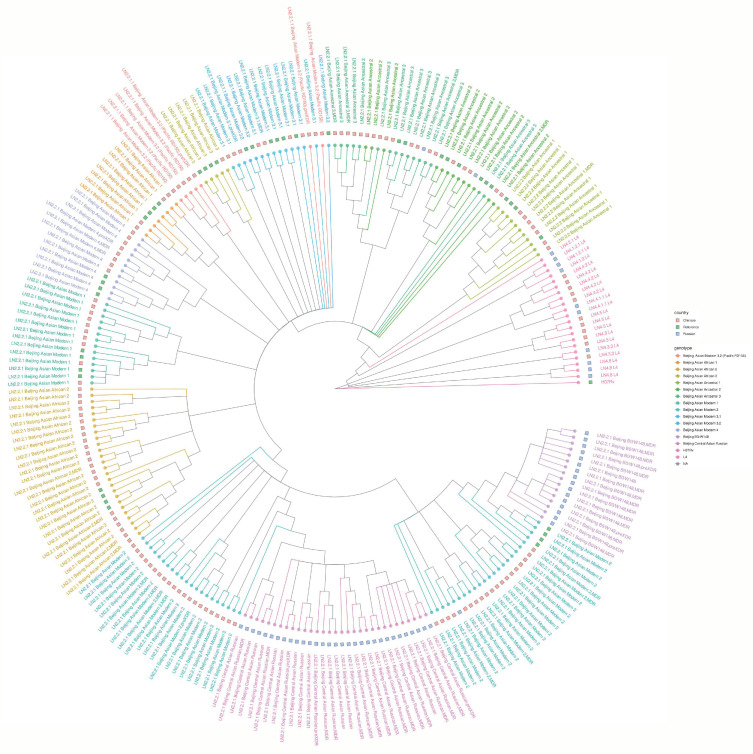
Maximum likelihood phylogenetic tree of Russian and Chinese pediatric *M. tuberculosis* strains. Subclade information and MDR/pre-XDR-TB status of the strains are included in their ID. Previously published representative genomes of different Beijing sublineages [16] were used as the references (see Materials and Methods for more details). See also Figure S1.

The ML ("maximum likelihood”) phylogenetic tree was constructed based on 13,302 polymorphic sites with reliable coverage of more than five reads per position (Figure 2). The Russian strains were assigned to Lineage 2 (Beijing genotype; *n* = 50 (83.3%)) and Lineage 4 (*n* = 10). Ten Russian Lineage 4 isolates included the following genotypes: Haarlem (*n* = 2), LAM (*n* = 1), Ural (*n* = 1), S (*n* = 2), and L4—unclassified (L4.8, *n* = 3, and L4.1.2, *n* = 1) (Figure 2, Table 1). The Chinese strains represented Lineage 2 (Beijing genotype; *n* = 126)) and Lineage 4 (*n* = 11). Of 126 Chinese Beijing genotype isolates, 26 belonged to the ancient/ancestral sublineage, and 100 belonged to the modern Beijing sublineages. Fifty Russian Beijing strains were classified as ancient/ancestral Beijing sublineages (*n* = 2) and modern Beijing sublineages (*n* = 48). The latter included epidemic and MDR-associated B0/W148-cluster, Central Asian/Russian, and Asian Modern 2 groups. Other Beijing modern subgroups were predominantly found in the Chinese dataset: 27 Asian African isolates, which were reported early [20,21], were assigned to the subgroups recently defined by Zhou et al. [22]: Asian Modern 1, Asian Modern 2, Asian Modern 3.1, Asian Modern 3.2 (Pacific RD150), and Asian Modern 4. Eleven Chinese L4 isolates included five L4.5 isolates, one LAM RD115 isolate, and five L4.4.2 isolates. Noteworthy, half of the Chinese L4 isolates belonged to L4.5, characterized by RD122 detection. Globally, this sublineage is a minor one, but it is known to be the only L4 sublineage endemic in China, while its South China origin was hypothesized [23].

The WGS-based phylogenetic analysis of all studied strains (Figure 2) showed a clear separation of the East Asian (L2) and Euro-American (L4) lineages. All L4 genomes of Lineage 4 are represented by the most distal branches of the tree. Clusters within L4 have significant bootstrap values, but basal branches are not supported (Figure 2 and Figure S1). Among the Beijing genotype strains (Lineage 2), the Beijing Ancestral 1 group is located distantly from all other L2 isolates, and all branches in this group are resolved. The remaining Beijing Ancestral genomes (Beijing Ancestral 2 and 3) form a common cluster (bootstrap value 100, Figure S1), where only some genomes have dichotomous branching. The main phylogenetic groups of Lineage 2.2.1 (Beijing modern) are supported by high bootstrap values (95–100%) (Figure 2 and Figure S1). Some of the internal branches within the modern Beijing group are not resolved, which probably indicates a different rate of evolution of some clusters within L 2.2.1. The majority of Russian strains were in two genetically compact groups, Beijing Central Asian/Russian and Beijing B0/W148 (neither was identified in the Chinese collection). On the whole, modern Beijing strains from China were more divergent and formed several genetic groups: Beijing Asian Modern 1, 2, 3, and 4, as well as Beijing Asia-Africa 1, 2, and 3.

We filtered out the synonymous SNPs, previously described phylogenetically informative markers for *M. tuberculosis* genotypes [24,25,26,27,28], drug resistance mutations, and PE/PPE genes. Among the remaining SNPs, we found only one additional SNP marker that appears to be novel for the Russian dataset. In addition to known *ndh* V18A and *mshA* N111S mutations, suggested as markers of S and Haarlem genotypes [24], we found an *ethA* A76D mutation in the studied S genotype strains. Its specificity should be further validated in the larger and geographically more diverse dataset.

### 2.2. Phenotypic Drug Resistance in M. tuberculosis Genotypes

In the Russian collection, twelve (19.7%) of sixty isolates were susceptible to both INH and RIF; thirty-eight (63.3%) isolates were MDR, and eight of them were resistant to INH, RIF, and FQ, i.e., they were pre-extensively drug-resistant (pre-XDR) (Table 2). The high proportions of MDR and pre-XDR isolates in the Russian dataset were found for both major Beijing subgroups: B0/W148 (94% and 19%) and Central Asian Russian (58% and 10%). The majority of B0/W148 strains were INH- and RIF-resistant (15/16), and three of them were pre-XDR, compared to the smaller rate in Central Asia/Russian (19/31) (*p* = 0.02) and non-Beijing strains (5/10) (*p* = 0.03) in the Russian collection. In the Chinese dataset, the majority of strains (95/137; 69.3%) were phenotypically susceptible, 27 (19.7%) were MDR, and 3.6% (7/27) of MDR were pre-XDR (Table 3). Chinese strains had MDR and pre-XDR among new TB patients (10.9% (13/119) and 3.4% (4/119), respectively) and previously treated patients (100% (14/14) and 21.4% (3/14), respectively). Ten of thirteen new cases had primary MDR-TB, since the isolates were collected in the Beijing Children’s Hospital before (5/10) or at the beginning (5/10) of treatment. Chinese isolates of the ancient Beijing (5/26), modern Beijing (20/100), and non-Beijing (2/11) groups did not differ significantly in MDR (*p* = 0.97) and pre-XDR (*p* = 0.78) rates. The MDR and pre-XDR rates in Chinese Beijing strains of modern (12/78) and ancient (0/26) sublineages also did not differ significantly in new cases (*p* = 0.136). Still, we note that MDR was absent in strains from newly-diagnosed Chinese children infected with ancient Beijing strains.

### 2.3. Genotypic Drug Resistance

#### 2.3.1. Distribution of Mutations Associated with Drug Resistance in *M. tuberculosis*

We identified mutations in 23 resistance genes that are known to be involved in resistance to anti-TB antibiotics (Table 4) [29,30,31,32,33]. The concordance between the presence of such mutations in phenotypically resistant strains was high (while phenotypic DST was a reference): 92.3% for RIF (DST: WGS; 65:60 isolates), 96.2% for INH (DST: WGS; 78:75 isolates), 97.4% for EMB (DST: WGS; 38:37 isolates), 93.5% for STR (DST: WGS; 77:72 isolates), 75.0% for FQs (DST: WGS; 20:15 isolates), and 85.7% (DST: WGS; 21:18 isolates). However, the pooled analytical performance of WGS data (sensitivities, specificities, positive predictive value (PPV), and negative predictive value (NPV)) varied significantly, depending on the number of genes, their allele variants, and the prevalence of resistance (Table 5).

The data are shown with 95% confidence intervals. The phenotypic method was used as a reference.

#### 2.3.2. Analysis of Mutations Profiles in Drug-Resistant Isolates

**Figure 3:**
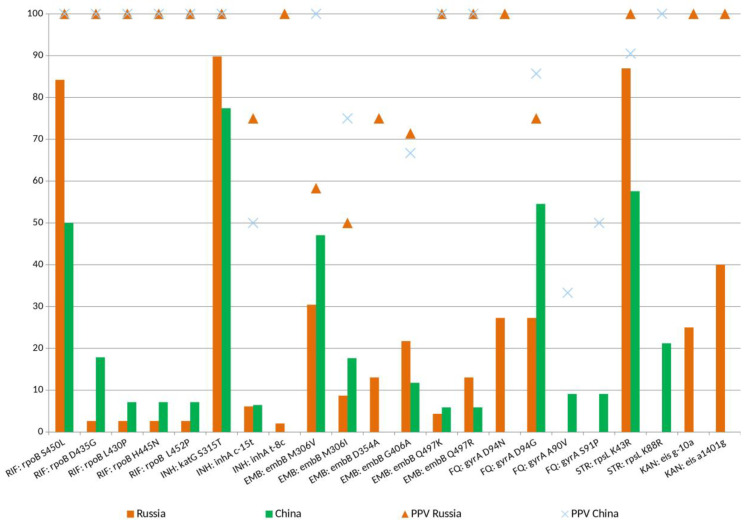
Frequency of main mutations in phenotypically resistant *M. tuberculosis*. Bars represent the specific relative frequency of each mutation among resistant isolates from Russian (orange) and Chinese (green) datasets. Triangle and cross marks locate PPV values for respective mutations to detect drug resistance in Russian and Chinese collections, respectively. RIF—Rifampicin, INH—Isoniazid, STR—Streptomycin, FQ—Fluoroquinolones, EMB—Ethambutol, KAN—Kanamycin.

Drug resistance in Russian and Chinese databases is driven by common sets of drug resistance-associated mutations (Figure 3). Chinese RIF-resistant (RR) strains had a wider variety of RIF-related mutations, and the major mutations were most common in both collections, but they differed in their prevalence. In particular, *rpoB* S450L accounted for 84.2% (32/38) and 51.9% (14/27) of RR Russian and Chinese isolates (*p* = 0.003). Out of 38 RR Russian strains, 35 had mutations, and they were found only in the *rpoB* gene (Table 6). Besides the canonical *rpoB* S450L associated with a high level of RR [34], other mutations in *rpoB* RRDR (rifampicin resistance determining region) were identified (L430P, D435G, H445N, and L452P), which were found in the Beijing Central Asian/Russian clade. We observed double changes in *rpoB* in Russian isolates (7/38; 18.4%), i.e., combinations of RRDR mutations and other *rpoB* mutations outside RRDR in phenotypically resistant isolates of the Central Asian Russian subtype (T399I, I480V, E761D, and R827C) and B0/W148 (L42V, R827L, and R827C). They may act as compensatory mutations in *rpoB* (Table 6), which provide a fitness benefit [24,27], or they may be neutral phylogenetic markers. Possibly compensatory mutations in *rpoC* or *rpoA* genes were not found in Russian RR strains. Chinese RR isolates had mutations in the *rpoB*: single mutations were in S450L (14/27), 51.9%), D435G (3/27, 11.1%), and L452P (1/27), and seven strains harbored double mutations in RRDR. Four Chinese MDR isolates had compensatory mutations: in *rpoC* (I491V) and *rpoB* (V170F, Q401R), in addition to changes in RRDR, and one isolate had a combination of *rpoB* V170F and *rpoC* I491V mutations. While any non-synonymous mutations in *rpoC* are considered as putatively compensatory, three *rpoC* mutations (A230V, P444S, and Y722C) were found in both datasets in RIF-sensitive strains and were not considered as associated with the RIF resistance phenotype (Table S1).

Mutations in the well established INH resistance genes and regulatory regions (*katG, inhA* promoter, or *ahpC* promoter) were found in INH-resistant isolates. *katG* S315T was found in 75.0% (36/48) and 67.4% (21/31) of Russian and Chinese INH-resistant strains, respectively, in similar frequencies (*p* = 0.48). When mutations in *inhA* mutations (S94A, L203L), its promoter region (*fabG1* c-15t, t-8c), and *ahpC* (c-52t and g-48a) were additionally considered, along with *katG* S315T, the rate of genotypically INH-resistant isolates increased to 91.7% and 77.4% in Russian and Chinese collections, respectively. Of three isolates with the *ahpC* SNPs, one carried a high-confident combination of mutations *katG* S315T, and *ahpC* g-48a, one had a non-confident mutation related to *katG* P232A and *ahpC* g-48a, and one had wild-type *katG*. Beijing B0/W148 had only *katG* S315T mutations alone. The discordant cases presented: (i) three INH-resistant isolates per each dataset without known mutations and (ii) two INH-susceptible isolates with *inhA* c-15t mutation. Of interest was the identification of mutations in *Rv2752*, which was previously suggested to play an indirect role in MDR tolerance [35]. However, *Rv2752* mutations were present in both phenotypically RIF/INH-resistant isolates with major mutations (*katG* S315T + *Rv2752c* H86Y+ *rpoB* S450L + *rpoB* Q401R), in an INH-resistant isolate (*katG* S315T + *Rv2752c* L487F), and in sensitive isolates (*Rv2752c* P101A, K357T, or R361G) (Table S1).

Generally, the discrepancies between phenotypic DST and genome-based detection of drug resistance could be due to unknown mechanisms of resistance and rare (as yet non-proven) resistance mutations. In this sense, more large-scale population-based studies worldwide are required to find and confirm such rare mutations to be associated with resistance. On the other hand, there is a possibility of errors in phenotypic drug susceptibility testing; unfortunately, live strains were not available for reculture and repeating DST.

The mutations associated with EMB resistance were detected in MDR isolates in Russian (29/38) and Chinese (19/27) datasets. Of 38 EMB-resistant isolates, 37 had *embB* mutations, mostly as single mutations (Table 6). M306V and M306I were common mutations in two collections and were detected in 40.9% (9/22) and 68.8% (11/16) of Russian and Chinese EMB-resistant strains, being more prevalent in the Russian collection, but not significantly (*p* = 0.09), perhaps due to small sample size. The presence of the *embB306* mutations in phenotypically EMB-susceptible MDR strains significantly decreased the specificity for the detection of drug resistance (Table 5). This phenomenon was first discovered in Russian EMB-susceptible MDR strains [36] and confirmed in a multicenter study [37], while *embB* mutations were suggested as a marker of broad drug resistance. Only one Chinese EMB-resistant isolate had a single mutation in *ubiA* (I179T). However, *ubiA* mutations were also detected in EMB-susceptible strains: *ubiA* V188A in the Russian MDR isolate and *ubiA* g-3787a in the Russian polyresistant isolate. In this view, the association of *ubiA* and EMB resistance is questionable.

PZA is an important first-line anti-TB drug, and although PZA DST data were not available for our collections, we looked at *pncA* mutations, which were highly diverse and scattered across the gene. Thirty MDR isolates had *pncA* mutations, of which only L120Q and Q141P were common for both datasets, and one Chinese isolate has *Rv3236*c c-365g. The frequency of *pncA* mutations in MDR-TB patients in both datasets did not differ significantly (20/38 of Russian and 10/27 of Chinese isolates; *p* = 0.2).

New WHO guidelines include fluoroquinolones, bedaquiline, and linezolid as key second-line drugs, whose resistances define the pre-XDR and XDR status of isolates [1,11]. In the studied collection, 20 isolates were FQ-resistant. Fourteen of them had mutations, either in *gyrA* (D94N, A90V, S91P) or *gyrB* (S447F). No mutations were found in the bedaquiline resistance gene *atpE* and linezolid resistance gene *rplC*. This is not unexpected, since the isolates were obtained before the wide use of bedaquiline and linezolid for TB treatment in Russia and China. Other substitutions possibly associated with bedaquiline resistance were found in two Chinese pre-XDR (*Rv1979c* V426I and *Rv1979c* a-130c) isolates, one Russian pre-XDR (*mmpL5* M655T) isolate, and in fully susceptible isolates (*mmpL5* V55M, *mmpL5* G246S, *mmpR (Rv0678),* Q51K, *Rv1979c* g-70a, and *mmpS5* g-74t).

Group C of the recommended anti-TB drugs includes delamanid, amikacin, streptomycin, and ethionamide. In our collections, mutations in the delamanid resistance genes (*fbiA*, *fbiC*, *ddn*, and *fgd1*) [38] were detected in Russian strains with *fgd1* K270M (Haarlem genotype of Lineage 4), as well as in two mono/polyresistant isolates (*ddn* R30S in Beijing Central Asian Russian isolate and *fbiA* I208V in an isolate of Lineage 4.1.2).

MDR strains (34/38 and 21/27) appear to have a higher potential to be resistant to STR than non-MDR strains (8/22 and 11/110) in both collections (*p* < 0.0001), but Russian and Chinese STR-resistant strains somewhat differed in the prevalence of major mutation: *rpsL* K43R was in 88.9% (40/45) of Russian and 59.3% (19/32) of Chinese collections (*p* = 0.003). The STR-resistant B0/W148 strains had double mutations: *rpsL* K43R + *whiB6* T51P. The *rpsL* K88R was in 21.9% (7/32) of Chinese strains. *gidB* G71E, *gidB* G34V, *gidB* D107G, or *Rv1258c* G363V mutations were present in some genetically distinct isolates without *rpsL* mutations, indicating that they are targets of selective pressure, which supports their possible role in STR resistance [39].

The drug resistance mutations, involving either *rrs* or *eis* genes associated with resistance to KAN, AMI, and CAP, were detected in low proportions among resistant isolates. In most cases, Russian KAN-resistant isolates had *rrs* a1401g and *eis* g-10a without additional mutations in other positions and genes. There were two sensitive isolates with *rrs* a1401g. Among nine resistants to KAN, AMI, and CAP, seven strains had single mutations in *rrs* a1401g, and two had no mutations in *rrs, eis,* and *tlyA*. Mutations causing resistance to KAN, AMI, and CAP all tend to be localized around *rrs* position 1400 because these drugs bind to a distinct region of the 16S rRNA molecule, known as the “A-site” (aminoacyl-tRNA site) [40]. Based on these positions, the KAN-, AMI-, and CAP-sensitive *rrs* mutants outside positions 1401, 1402, or 1484 were considered as non-associated with resistance.

Ethionamide (ETH) DST data were available only for Russian strains. Comparison with WGS data for 28 Russian ETH-resistant strains revealed only partial concordance with DST. It can be explained by the presence of partial cross-resistance to INH in *inhA* mutations that affect the activity of InhA reductase and its binding properties with toxic adducts. Mutations in the *inhA* promoter were found in ETH/INH-susceptible and INH-resistant strains (c-15t), as well as in two ETH-resistant strains with *inhA* t-8c and L203L. Detection of these mutations in both resistant and susceptible strains may reflect the role of *inhA* promoter mutations, leading to an increase in gene expression, thus decreasing the toxic effect of INH and ETH [41]. Most of the identified mutants had *ethA* frameshift and nonsense mutations in one-third of isolates (3/9).

## 3. Discussion

The MDR-TB epidemics in China and Russia differ in key epidemiological aspects. In Russia, despite the overall decline in TB incidence, the proportion of primary MDR-TB is increasing (from 9.5% in 2005 to 34.2% in 2019), which indicates the active transmission of MDR strains in the population, including children [42,43]. The MDR rate in children with TB increased four times and reached 45.8% in 2019 [3]. In contrast, in China, according to WHO estimates, the MDR-TB rate among new cases was 5.7% (4.5–7.0%) [44], with a rising trend between 2012 and 2018 [45,46], while the prevalence of MDR-TB in childhood TB was stable, at 5.6% in 2008 to 2018 [47]. According to the most recent WHO estimation, the prevalence of MDR-TB in childhood TB in China in 2021 was 3.4% (3.3–3.5) [1]. Studies on drug resistance characteristics of pediatric TB cases have been rare in China and Russia, partly due to the known difficulty of obtaining clinical isolates from pediatric patients. In this study, we report the analysis of phenotypic and molecular patterns of drug resistance of pediatric *M. tuberculosis* strains. Based on whole genome sequencing data, we obtained the genotypic identification of strains and the complete set of mutations conferring drug resistance according to the latest WHO catalog [16]. We analyzed the phenotypic and genotypic profiles of *M. tuberculosis* strains from children using the new MDR/XDR definitions [1,11].

The majority of strains from the Russian cohort (63.6%) and 19.7% of the Chinese cohort were phenotypically MDR. These values are greater than the reported rates of MDR-TB in the pediatric populations in the studied countries [3,48,49]. The same was true for pre-XDR resistance (13.3% of Russian and 3.6% of Chinese isolates). On the other hand, a similarly designed Russian adult study also showed a high percentage of MDR (73.3%) in Russian patients with spinal TB that were also predominantly infected with Beijing genotype trains [50]. Our previous pediatric study in China reported a high MDR rate of 22%, including 11.7% in new cases, and an extremely high 56.5% in previously treated cases) [48]. Indeed, all previously treated Chinese patients were infected with MDR strains, although 91.2% of the cohort consisted of never or shortly treated patients. Finally, a speculative, but still plausible, explanation for both cohorts may be that drug resistance was not acquired during treatment but the children (especially, young children) were infected within their families/households from their chronic relatives who had multiple retreatment courses.

Based on WGS data, we demonstrated that Beijing strains were predominant in both collections followed by Euro-American lineage. However, the phylogenetic analysis demonstrated a striking difference in the Beijing structures, especially Asian Modern groups, between the two countries. The strongly MDR and transmissible Beijing B0/W148 strains were identified in Russia, and the Beijing Central Asian/Russian clade was predominant in Russia on the whole [24,49]. On the other hand, only two isolates of the Beijing Asian Ancestral and one isolate of Beijing Asian Modern 2 subtype were found in the Russian collection, reflecting that most of the isolates were from the European part of Russia [24,39], where Ancestral Beijing isolates are less common than in Siberia [51,52]. There were eleven (three Ancestral and eight Modern) major Beijing genotype subclades in the studied Chinese pediatric cohort. This is consistent with the distribution of isolates from the national drug resistance survey in adult TB cases in China [22,53]. Within modern Beijing strains, we observed large and heterogeneous branches, known as Asian African 1, 2, and 3, as well as recently classified Asian Modern groups. The Central Asian Russian subclade was located within Chinese Asian Modern 2 on the phylogenetic tree (Figure 2 and Figure S1). Compared to the diverse Modern Beijing isolates from China, Russian Beijing B0/W148 isolates formed a monophyletic group, consistent with a single relatively recent expansion of this subtype in Russia with hypothesized origin in Siberia in the 1950s [49], which was recently supported by WGS analysis [54].

Since the studied Russian isolates were obtained from surgical specimens, we compared our data to a similar adult study carried out in Russia on patients with spinal TB admitted to the same referral hospital in St. Petersburg [50]. In that study by Vyazovaya et al. [50], the Beijing genotype was highly predominant in extrapulmonary TB patients, at 75%, which was much higher compared to pulmonary TB patients (average 45–50% in different Russian studies [49]). The high proportion (28.0%) of the B0/W148 subtype was also noted in that adult study of spinal TB, which was higher compared to pulmonary TB studies in Russia [50]. These findings on the increased prevalence rate of the Beijing genotype and its epidemic cluster B0/W148 in adult patients with extrapulmonary TB in Russia are similar to our data on children with extrapulmonary TB.

As one of the main objectives of this study was to compare adult versus pediatric strains at within-country level, we looked at the comparable studies on the general population that could be used as an adult reference with regard to our study. In the case of Russia, this was the above-cited study [50] that targeted the same kind of patient cohort as in this pediatric study: country-wide bone TB surgery cases admitted at the same referral hospital in St. Petersburg. With regard to China, the study by Zhou et al. [22] targeted the same kind of patient cohort as in this pediatric study: a countrywide survey when representative strains were selected based on their genotypes, drug susceptibility patterns, and origin. Thus, we compared the prevalence of the genotypes identified in this pediatric study to their prevalence in these Chinese and Russian studies (Table 7 and Table 8; Figures S2 and S3). The main sublineages and subtypes of the dominant Beijing genotype and the main endemic non-Beijing clades were present in both adults and children in Russia (Table 8). In particular, the Russian epidemic clone B0/W148 was found in almost the same percentage in adults (28.6%) and children (26.7%). The only more prominent difference was in the prevalence rate of the most widespread and prevalent Russian Beijing Central Asia Russian clade, which was more frequent in children compared to adults (51.7% vs. 37.1%), but at a non-significant level (*p* = 0.07). In the Chinese collection, all main Beijing subtypes and the main Chinese non-Beijing clade L4.5 (RD122) were present in both adult and children groups (Table 7). Unlike the Russian collection (which could be affected by the small sample size), some differences were statistically significant when Chinese groups were compared. Regarding the Beijing family, Beijing Asian African 3 was more prevalent among adults (7.4% vs. 0.7%), and Beijing Asia Modern 2 was more prevalent in children (29.2% vs. 17.1%). As concerns non-Beijing L4.5 sublineages, they are more prevalent among adults (9.5% vs. 3.6%). On the whole, at the large-scale level, when all Beijing subtypes were merged into modern and ancestral groups, the Beijing modern sublineage was more prevalent in Chinese children (73.0% vs. 61.7%; *p* = 0.017). Whether these differences reflect particular features related to differential transmission/pathogenicity of certain strains or lineages in either adults or children should be further investigated through a systemic approach and epidemiological prospective study.

The finding of different levels of DR could be explained in part by the more active spread of certain successful genotypes [20,43] associated with MDR and XDR in Russia. Indeed, 26.7% of the studied Russian isolates belonged to the Beijing B0/W148. The prevalence of the common *rpoB* S531L + *katG* S315T double mutation was higher in Russian isolates (31/38 Russian versus 11/27 Chinese MDR isolates; *p* = 0.002), particularly in Russian B0/W148 (81.3%; 13/16). The findings on the Russian MDR cohort likely reflect the clonal expansion of certain epidemic strains bearing low fitness-cost mutations (*rpoB* S450L, *katG* S315T, and *rpsL* K43R) [24,55]. This is reflected in the higher MDR prevalence in the Beijing B0/W148 subtype (93.8%) compared to Central Asian Russian (61.3%) (*p* = 0.02) and non-Beijing Russian strains (40.0%) (*p* = 0.03). Chinese Beijing Asian Ancestral (MDR rate 18.5%), total Modern Beijing (20.0%), and non-Beijing (18.2%) strains did not differ significantly in MDR (*p* = 0.97) and pre-XDR (*p* = 0.78) prevalence rates, which indirectly confirms the previously obtained data about the absence of major outbreaks of MDR-TB in China, which are associated with any Beijing sublineage [4,6,22].

We expected to find high frequencies of compensatory mutations in Russian MDR strains reported in other studies, such as *rpoB* E761D in the successful clone of the Beijing Central Asian subtype in Russia [24], which was found in our data set, but only in one isolate [24]. Potentially compensatory *rpoB* mutations were observed in Russian epidemic MDR-TB strains, as well as in all different lineages of Chinese strains. The total number of compensatory mutations in RR isolates from pediatric TB cases did not differ, but they were less frequent (18.4% in Russian and 14.8% in Chinese datasets) than those described for clinical isolates from adult TB patients (47% [24] and 30% [22,56]). Compensatory mutations in Russian isolates were found only in *rpoB*, and, in the Chinese isolates, they were in *rpoB* and *rpoC*. We found only three cases of known compensatory mutations to INH in relation to *ahpC*. These findings indicate that these strains with compensatory mutations had not yet become widespread, and not all compensatory mutations are important for the spread of resistant mutants [57]. It may be that the major resistance mutations selected in the Russian epidemic successful strains have low-fitness cost, and, therefore, a selection of additional compensatory mutations would be redundant from the evolutionary viewpoint.

Despite different patterns and structures of drug resistance in the studied countries and their levels in our datasets, we found a strong correlation between MDR and resistance to other first-line drugs (EMB, PZA). We examined the contribution of the different confidence-graded mutations to overall resistance in the studied datasets. Our data confirmed the high performance of WGS for the detection of rifampicin and isoniazid resistance, but for the remaining first-line drugs and FQ, the sensitivities and specificities were lower, possibly due to the insufficient knowledge of molecular mechanisms and genes involved in the development of resistance to STR, EMB, and PZA, as well as lack of standardization in methodologies and drug concentrations for phenotypic tests for PZA.

## 4. Materials and Methods

### 4.1. Strain Collection

In China, the study was conducted on children aged < 17 years from all over the country admitted to Beijing Children’s Hospital, China, and this study included children who were diagnosed with TB between 2005 and 2016. In Russia, the study was conducted on children aged < 16 years old admitted to the clinics of the St. Petersburg Research Institute of Phthisiopulmonology from all regions of the country for surgical treatment in 2006–2020. In total, 61 Russian and 141 Chinese isolates were collected in the bacteriology laboratories at the above hospitals. Five *M. bovis* were identified and excluded from the analysis. Therefore, the final collection included 197 *M. tuberculosis* isolates recovered from 197 pediatric TB cases from China (*n* = 137) and Russia (*n* = 60). The majority of *M. tuberculosis* isolates were from male patients (65.6%; 39/60 Russian and 59.1%; 81/137 Chinese patients).

The Chinese cohort included *M. tuberculosis* isolates from pediatric patients with newly-diagnosed TB (86.9%; 119/137) and who were previously treated (10.2%; 14/137). Clinical specimens were collected predominantly before treatment (43.8%; 60/137) and at the beginning of the intensive phase of anti-TB therapy (47.4%; 65/137).

The Russian cohort consisted of newly-diagnosed TB patients who were in the continuation phase of TB treatment (they were mostly receiving antibiotic therapy for up to 2–4 weeks, and no more than 1–2 months of the intensive phase), which included surgical treatment for all 60 patients. All Russian isolates were recovered from surgical specimens (Figure 1). Under the principles of treatment of the bone and joint tuberculosis (BJTB) applied in Russia [58], the indication for surgery was the presence of inflammatory (abscesses), neurological (spinal cord compression), or orthopedic complications (loss of support or deformities caused by instability of the bone segment). In these cases, surgical treatment was carried out after 2–4 weeks from the initiation of anti-tuberculosis therapy. The exception was patients with complicated bronchopulmonary lesions, in whom surgical treatment of the BJTB lesion zones was performed no earlier than 2 months from the start of complex chemotherapy. In the event of neurological disorders, intervention on the spine was irrespective of already initiated chemotherapy to minimize the risk of irreversible paralysis.

The Chinese isolates were cultured from body fluid samples, mainly including bronchoalveolar lavage fluid (40.1%), gastric aspirates (27.7%), sputum (10.4%), cerebrospinal fluid (8.8%), and other sources (13.1%). Russian *M. tuberculosis* isolates were cultured from surgical specimens of vertebrae (32), bones (24), joints (3), and bronchus (1).

### 4.2. Drug Susceptibility Testing

The study included retrospective DST data of Russian and Chinese strains obtained by the method of proportions on the Loewenstein-Jensen medium or Middlebrook 7H10 medium and by the method of absolute concentrations on the Loewenstein-Jensen medium according to the WHO recommendations in China [59] and Orders of the Russian Ministry of Health in Russia [60,61].

Drug susceptibility testing (DST) was performed for six anti-TB drugs in China (Isoniazid (INH), Rifampicin (RIF), Ethambutol (EMB), Streptomycin (STR), Ofloxacin (OFL), and Kanamycin (KAN)) and nine drugs (INH, RIF, EMB, STR, Moxifloxacin (MOX), OFL, Levofloxacin (LEV), Ethionamide (ETH), KAN, Capreomycin (CAP), and Amikacin (AMI)) in Russia.

Critical concentrations of the drugs were as follows: INH 0.2 mg/L, RIF 40 mg/L, EMB 2 mg/L, STR 4 mg/L, OFL 2 mg/L, KAN 30 mg/L, ETH 30 mg/L, CAP 40 mg/L, AMI 40 mg/L [59]. The phenotypic drug susceptibility testing method was used as a reference to assess the sensitivity, specificity, positive-predictive value, and negative-predictive value of WGS data.

### 4.3. Whole Genome Sequencing

Genomic DNA was extracted from *M. tuberculosis* cultures by the cetyltrimethylammonium bromide-lysozyme (CTAB) method [62]. In Russia, WGS was performed on the Illumina HiSeq4000 platform using NEBNext Ultra, MiSeq Reagent v3, and PhiX Control v3 kits (Illumina). DNA libraries were prepared using ultrasound DNA fragmentation and NEBNext Ultra DNA Library Prep Kit for Illumina (New England Biolabs, Frankfurt, Germany). In China, WGS was performed on the Illumina MiSeq platform in Novogene Bioinformatics Technology Co. Ltd. (Beijing, China) DNA libraries, which were prepared using Nextera XT kits (Illumina, San Diego, CA, USA). The raw reads were submitted to the NCBI SRA archive under accession number PRJNA786957.

### 4.4. Bioinformatics and Phylogenetic Analysis

All read pairs were processed by CutAdapt [63] for quality trimming and removal of adapter sequences. Mapping of short reads to the reference gene *M. tuberculosis* H37Rv (NC 000962) was performed using the Burrows-Wheeler aligner [64]. The extraction of sequences from the mapped genome with the calculation of the coverage of each position was carried out with SamTools [65].

The concatenated sequence alignment for the phylogenetic analysis was created with consideration of minimum thresholds of five reads in both forward and reverse orientation, five reads calling the SNP with a Phred score > 20, and 75% SNP frequency. All SNPs within drug resistance genes with promoters and highly variable genes (PE, PPE) were excluded from the analysis. The input data for the analysis was composed of 241 sequences, containing a total of 13,302 nucleotide sites. Among these sites, the number of parsimony informative sites was 2551. The model finder selected the TVM+F model as the most suitable one based on the Bayesian Information Criterion (BIC). The consensus tree is constructed from 1000 bootstrap trees by IQ-TREE [66].

Visualization of the phylogenetic tree was performed by the ggtree r-package [67]. *M. tuberculosis* strains were grouped by lineage and subgroups using the classifications by Coll et al. [68], Shitikov et al. [21], and Napier et al. [69]. For Beijing sublineages, we additionally used a recent classification of Asian Modern clades by Zhou et al. [22] along with their WGS data (NCBI: PRJNA573798) as reference. We used 4 reference genomes from [22] per each of the newly determined 11 Beijing sublineages to better phylogenetically locate Chinese isolates. We followed this course of analysis because previous classifications [21,68,69] clearly subdivided the Beijing isolates into large-scale modern and ancient sublineages, but they failed to classify a large group of Chinese isolates. In contrast, the use of the Chinese reference genomes [22] permitted us to assign Chinese strains to the particular sublineages described by Zhou et al. [22].

Drug resistance mutations were assigned based on the WHO catalogue [16].

### 4.5. Statistical Analyses

All data were analyzed with the STATISTICA 10.0 software package (StatSoft Inc., Tulsa, OK, USA). For each mutation, a contingency table of binary phenotypes and the presence or absence of the mutation were made to calculate sensitivity, specificity, positive-predictive value, and negative-predictive value for six anti-TB drugs (RIF, INH, ETB, STR, OFL, and KAN). Pearson’s chi-squared test and Fisher’s exact test were performed to determine the significance of categorical variables. The differences were considered statistically significant when *p* < 0.05.

### 4.6. Limitations

Russian isolates were cultured from surgical samples of pediatric TB patients who were receiving antibiotic therapy for up to 2 months of the intensive phase, and then, for medical reasons, were referred for surgical treatment as a continuation phase. Although these children were newly diagnosed TB cases and most of them received chemotherapy during 2–4 weeks before surgery, some of them were receiving anti-TB drugs for 1 to 2 months, and in this case, drug resistance could potentially be acquired by mycobacteria in case of the suboptimal treatment regimen. This presented a certain limitation in the evaluation of primary drug resistance in this cohort.

The study included retrospective DST data from Russian and Chinese collections. The methods recommended at that time in Russia were the proportion method on the Loewenstein-Jensen medium and the Middlebrook 7H10 medium and the absolute concentration method on the Loewenstein-Jensen medium, according to the recommendations for Russia Orders of Ministry of Health of the Russian Federation [60,61]. The methods recommended at that time in China were the absolute concentration method on the Loewenstein-Jensen medium and changed to the microplate method in 2016, according to WHO recommendations [59]. This was a certain limitation for the comparative evaluation of phenotypic and genotypic drug resistance of the studied strains.

## 5. Conclusions

By combining WGS and phenotypic susceptibility data for 197 isolates, we identified drug resistance patterns of *M. tuberculosis* isolates from children in high-burden MDR-TB countries, China and Russia. *M. tuberculosis* strains from children display molecular patterns of drug resistance shaped by locally endemic phylogenetic clades. The genotypes of clinical isolates of *M. tuberculosis* in children from Russia and China were similar to those described in adult patients in the same countries. However, the Beijing Central Asian/Russian clade was more prevalent in Russian children compared to adults, and in the Chinese collection, certain genotype groups were significantly more prevalent in either pediatric (Beijing modern sublineage) or adult (L4.5 sublineage) cohorts. A new prospective study to confirm these differences and gain insight into the underlying reasons is warranted.

Regarding drug resistance determinants, MDR in the Russian collection was mainly (28/38; 74%) caused by mutations that do not adversely affect the viability and transmissibility (*rpoB* S450L + *katG* S315T + *rpsL* K43R), which indicates a large epidemic reservoir of MDR *M. tuberculosis* in Russia. In turn, only one-third of Chinese MDR isolates (8/27; 30%) harbored such a combination of mutations. The compensatory mutations in MDR strains from children were detected in some isolates, but they were not widespread. This situation may reflect unknown compensatory mechanisms that emerged during the early development of drug resistance due to inadequate chemotherapy before the widespread introduction of DST in Russia and China. The molecular mechanisms of adaptation of *M. tuberculosis* to anti-TB treatment are not unique to the pediatric population, but they reflect the general situation with the spread of drug-resistant TB in Russia and China.

## Figures and Tables

**Table 1:** Population structure of pediatric *M. tuberculosis* strains from China and Russia.

Lineage	Genotype/Subtypes *	Russia (*n* = 60)	China (*n* = 137)
L2.2.2	Beijing Asian Ancestral 1	0	7 (5.1)
L2.2.1	**Beijing Asian Ancestral 2**	**1 (1.7)**	**8 (5.8)**
L2.2.1	**Beijing Asian Ancestral 3**	**1 (1.7)**	**11 (8.0)**
L2.2.1	Beijing Asian African 1	0	4 (2.9)
L2.2.1	Beijing Asian African 2	0	22 (16.1)
L2.2.1	Beijing Asian African 3	0	1 (0.7)
L2.2.1	Beijing Asian Modern 1	0	10 (7.3)
L2.2.1	**Beijing Asian Modern 2**	**1 (1.7)**	**40 (29.2)**
L2.2.1	Beijing Asian Modern 3.1	0	8 (5.8)
L2.2.1.1	Beijing Asian Modern 3.2 (Pacific RD150)	0	7 (5.1)
L2.2.1	Beijing Asian Modern 4	0	8 (5.8)
L2.2.1	Beijing B0/W148	16 (26.7)	0
L2.2.1	Beijing Central Asian Russian	31 (51.7)	0
L4.1.2		1 (1.7)	0
L4.1.2.1	Haarlem	2 (3.3)	0
L4.2.1	Ural	1 (1.7)	0
L4.3.3	**LAM RD115**	**1 (1.7)**	**1 (0.7)**
L4.4.1.1	S	2 (3.3)	0
L4.4.2		0	5 (3.6)
L4.5	RD122	0	5 (3.6)
L4.8		3 (5.0)	0

* Genotype/subtypes are provided if the correlation between the SNP barcode and genotype name is known. Genotypes shared by strains from the two countries are in bold.

**Table 2:** Absolute numbers and percentage of isolates resistant to individual drugs and their combinations, stratified by genotype, in Russia. *The percentage is shown in italic*.

Genotype and Subtypes, Russian Collection	INH	RIF	EMB	STR	OFL	KAN	MDR	Pre-XDR
Beijing total	42/50; *84*	33/50; *66*	20/50; *40*	40/50; *80*	8/50; *16*	16/50; *32*	33/50; *66*	6/50; *12*
Beijing ancestral	1/2; *50*	0/2; *0*	0/2; *0*	1/2; *50*	1/2; *50*	0/2; *0*	0/2; *0*	0/2; *0*
Beijing modern other	1/1; *100*	0/1; *0*	0/1; *0*	0/1; *0*	0/1; *0*	0/1; *0*	0/1; *0*	0/1; *0*
Beijing modern B0/W148	16/16; *100*	15/16; *94*	10/16; *63*	16/16; *100*	3/16; *19*	8/16; *50*	15/16; *94*	3/16; *19*
Beijing modern Central Asian Russian	24/31; *77*	18/31; *58*	10/31; *32*	23/31; *74*	4/31; *13*	8/31; *26*	18/31; *58*	3/31; *10*
Non-Beijing total	6/10; *60*	4/10; *40*	2/10; *20*	5/10; *50*	2/10; *20*	4/10; *40*	4/10; *40*	2/10; *20*
ALL Russian	48/60; 80	37/60; *62*	22/60; *37*	45/60; *75*	10/60; *17*	20/60; *33*	37/60; *62*	8/60; *13*

**Table 3:** Absolute numbers and percentage of isolates resistant to individual drugs and their combinations, stratified by genotype, in China. *The percentage is shown in italic*.

Genotype and Subtypes, Chinese Collection	INH	RIF	EMB	STR	OFL	KAN	MDR	Pre-XDR
Beijing total	27/126; *21*	25/126; *20*	15/126; *12*	30/126; *24*	9/126; *7*	0/126; *0*	25/126; *20*	6/126; *5*
Beijing ancestral	7/26; *27*	5/26; *19*	5/26; *19*	8/26; *31*	2/26; *8*	0/0; *0*	5/26; *19*	1/26; *4*
Beijing modern, other	20/100; *20*	20/100; *20*	10/100; *10*	22/100; *22*	7/100; *7*	0/100; *0*	20/100; *20*	5/100; *5*
Beijing modern, B0/W148	0/0; *0*	0/0; *0*	0/0; *0*	0/0; *0*	0/0; *0*	0/0; *0*	0/0; *0*	0/0; *0*
Beijing modern, Central Asian Russian	0/0; *0*	0/0; *0*	0/0; *0*	0/0; *0*	0/0; *0*	0/0; *0*	0/0; *0*	0/0; *0*
Non-Beijing total	3/11; *27*	2/11; *18*	2/11; *18*	2/11; *18*	1/11; *9*	1/11; *9*	2/11; *18*	1/11; *9*
ALL Chinese	30/137; *22*	27/137; *20*	17/137; *12*	32/137; *23*	10/137; *7*	1/137; *1*	27/137; *20*	7/137; *5*

**Table 4:** Resistance genes with mutations detected in WGS data in phenotypically resistant isolates.

Gene Name	*Rv* Number	Associated Antibiotic Resistance
*gyrB*	*Rv0005*	moxifloxacin, ofloxacin, levofloxacin
*gyrA*	*Rv0005*	moxifloxacin, ofloxacin, levofloxacin
*rpoB*	*Rv0667*	rifampicin
*rpoC*	*Rv0668*	rifampicin
*rpsL*	*Rv0682*	streptomycin
*mmpR*	*Rv0678*	bedaquiline, clofazimine
*rrs*	*Rvnr01*	streptomycin, amikacin, kanamycin, capreomycin
*rrl*	*Rvnr02*	linezolid, capreomycin
*fabG1*	*Rv1483*	isoniazid, ethionamide
*inhA*	*Rv1484*	isoniazid, ethionamide
*ndh*	*Rv1854c*	ethionamide
*katG*	*Rv1908c*	isoniazid
*furA*	*Rv1909c*	isoniazid
*pncA*	*Rv2043c*	pyrazinamide
*eis*	*Rv2416c*	kanamycin
*ahpC*	*Rv2428*	isoniazid
*whiB7*	*Rv3197A*	streptomycin, kanamycin, amikacin
*aftA, embC*	*Rv3792,Rv3793*	ethambutol
*embA*	*Rv3794*	ethambutol
*embB*	*Rv3795*	ethambutol
*ubiA*	*Rv3806c*	ethambutol
*ethA*	*Rv3854c*	ethionamide
*gid B*	*Rv3919c*	streptomycin

**Table 5:** Performance characteristics of the molecular detection of drug resistance.

Drugs	Country	Sensitivity	Specificity	PPV
Rifampicin	Russia	0.89 (0.75–0.96)	1.00 (0.85–1.00)	1.00 (0.90–1.00)
	China	0.93 (0.77–0.99)	0.95 (0.89–0.99)	0.84 (0.66–0.95)
Isoniazid	Russia	0.98 (0.90–0.99)	0.92 (0.61–1.00)	0.98 (0.89–1.00)
	China	0.96 (0.79–0.99)	0.98 (0.93–0.99)	0.97 (0.79–0.99)
Ethambutol	Russia	0.95 (0.77–0.99)	0.84 (0.69–0.94)	0.78 (0.58–0.91)
	China	1.00 (0.80–1.00)	0.98 (0.93–0.99)	0.85 (0.62–0.97)
Streptomycin	Russia	0.87 (0.74–0.95)	0.93 (0.68–0.99)	0.98 (0.87–0.99)
	China	0.91 (0.76–0.98)	0.97 (0.92–0.99)	0.91 (0.76–0.98)
Fluoroquinolones (e.g., Levofloxacin, Ofloxacin, Moxifloxacin)	Russia	0.55 (0.23–0.83)	0.98 (0.89 + 0.99)	0.85 (0.42–0.99)
	China	0.73 (0.39–0.94)	0.99 (0.95–0.99)	0.89 (0.52–0.99)
Kanamycin, Amikacin, Capreomycin	Russia	0.90 (0.68–0.98)	1.00 (0.91–1.00)	1.00 (0.81–1.00)
	China	0.50 (0.01–0.98)	1.00 (0.97–1.00)	1.00 (0.08–1.00)

**Table 6:** Drug resistance alleles and their combinations in the studied isolates from Russia and China.

Drug	Mutations and Combinations	Russia *n* (%)	China *n* (%)
Rifampicin	No. of Rifampicin resistant isolates:	38	27
	*rpoB* V170F + *rpoC* I491V		1 (3.7)
	*rpoB* L430P		1 (3.7)
	*rpoB* L430P + *rpoB* D435G		1 (3.7)
	*rpoB* Q432P		1 (3.7)
	*rpoB* D435G		4 (14.8)
	*rpoB* H445N + *rpoB* L430P	1 (2.6)	
	*rpoB* H445N + *rpoB* H445R		1 (3.7)
	*rpoB* H445N + *rpoB* L452V		1 (3.7)
	*rpoB* H445N + *rpoB* D435G	1 (2.6)	
	*rpoB* S450L	26 (68.4)	12 (44.4)
	*rpoB* S450L + *rpoB* L42V	1 (2.6)	
	*rpoB* S450L + *rpoB* T399I	1 (2.6)	
	*rpoB* S450L + *rpoB* I480V	1 (2.6)	
	*rpoB* S450L + *rpoB* E761D	1 (2.6)	
	*rpoB* S450L + *rpoB* Q401R + *Rv2752c* H86Y		1 (3.7)
	*rpoB* S450L+ *rpoC* I491V		1 (3.7)
	*rpoB* S450L + *rpoB* R827C	1 (2.6)	
	*rpoB* S450L + *rpoB* R827L	1 (2.6)	
	*rpoB* L452P	1 (2.6)	1 (3.7)
	*rpoB* L452P + *rpoB* N437D		1 (3.7)
	No known mutations	3 (7.9)	1 (3.7)
Isoniazid	No. of Isoniazid resistant isolates:	48	30
	*katG* S315T	38 (79.2)	22 (73.3)
	*katG* S315T + *inhA* c-15t	2 (4.2)	
	*inhA* c-15t + *katG* A109+ *inhA* S94A	1 (2.1)	
	*katG* S315T + *inhA* L203L	2 (4.2)	
	*katG* S315T + *inhA* t-8c	1 (2.1)	
	*katG* S315T + *Rv2752c* L487F	1 (2.1)	
	*katG* S315T + *ahpC g-48a*	1 (2.1)	
	*ahpC* c-52t	1 (2.1)	
	*katG* S315T + *katG* c-441t		1 (3.3)
	*katG* P232A + *ahpC g-48a*		1 (3.3)
	*katG* F129S		1 (3.3)
	*katG* S315T + *Rv2752c* H86Y		1 (3.3)
	*inhA* c-15t + *katG F368L*		1 (3.3)
	*inhA L203L*		1 (3.3)
	*inhA* c-15t		1 (3.3)
	No known mutations	2 (4.2)	2 (6.6)
Ethambutol	No. of Ethambutol resistant isolates:	22	16
	*embB* M306V	5 (22.7)	7 (43.8)
	*embB* M306V + *embB* G406A	1 (4.5)	1 (6.3)
	*embB* M306V + *embC* c-1753t + *embA* c-12t	1 (4.5)	
	*embB* M306I	2 (9.1)	3 (18.8)
	*embB* S347I	1 (4.5)	
	*embB* D354A	3 (13.6)	
	*embB* G406A	3 (13.6)	1 (6.3)
	*embB* G406A + *embA* G5S	1 (4.5)	
	*embB* Q497K	1 (4.5)	1 (6.3)
	*embB* Q497R	2 (9.1)	1 (6.3)
	*embB* Q497R + *embC* A931T	1 (4.5)	
	*embB* T581A		1 (6.3)
	*ubiA* I179T		1 (6.3)
	No known mutations	2 (9.1)	0
Fluoroquinolones	No. of Fluoroquinolones resistant isolates:	9	11
	*gyrA* A90V		1 (9.1)
	*gyrA* S91P		1 (9.1)
	*gyrA* D94N	2 (22.2)	
	*gyrA* D94N + *gyrA* R578Q	1 (11.1)	
	*gyrA* D94G	3 (33.3)	5 (45.5)
	*gyrB* S447F	1 (11.1)	
	No known mutations	3 (33.3)	3 (27.3)
Streptomycin	No. of Streptomycin resistant isolates:	45	32
	*rpsL* K43R	24 (53.3)	17(53.1)
	*rpsL* K43R+ *whiB6* T51P	16 (35.5)	
	*rpsL* K43R+ *rrs* c517t		1(3.1)
	*rpsL* K43R + *whiB6* A99V		1(3.1)
	*rpsL* K43R + *whiB6* R107C	1 (2.2)	
	*rpsL* K88R		7 (21.9)
	*gidB* G71E		1 (3.1)
	*gidB* G34V		1 (3.1)
	*Rv1258c* G363V		1 (3.1)
	No known mutations	4 (8.8)	3 (9.3)
Kanamycin	No. of Kanamycin resistant isolates:	20	1
	KAN: *eis* g-10a	5 (25.0)	
	KAN: *eis* c-12a	1 (5.0)	
	KAN: *eis* c-14t + whiB6 g-42t	1 (5.0)	
	KAN: *eis* g-37t + whiB6 g-42t	1 (5.0)	
	KAN: *eis* g-37t	1 (5.0)	
	KAN: *rrs* a1401g	8 (40.0)	
	KAN: *whiB7* a-116g	1 (5.0)	
	No known mutations	2 (10)	1 (100)

**Table 7:** Comparison of pediatric versus adult *M. tuberculosis* populations from China (see also Figure S2).

Lineage	Genotype/Subtypes *	China Adult [20], *Number*	China Adult [20], %	China Children, This Study *Number*	China Children, This Study %	*p* **
L2.2.2	Beijing Asian Ancestral 1	11	2.6	7	5.1	0.16
L2.2.1	Beijing Asian Ancestral 2	14	3.3	8	5.8	0.2
L2.2.1	**Beijing Asian Ancestral 3**	36	8.6	11	8.0	0.8
L2.2.1	Beijing Asian African 1	21	5.0	4	2.9	0.3
L2.2.1	**Beijing Asian African 2**	63	15.0	22	16.1	0.7
L2.2.1	**Beijing Asian African 3**	31	7.4	1	0.7	**0.02**
L2.2.1	Beijing Asian Modern 1	14	3.3	10	7.3	0.052
L2.2.1	**Beijing Asian Modern 2**	72	17.1	40	29.2	**0.002**
L2.2.1	Beijing Asian Modern 3.1	11	2.6	8	5.8	0.08
L2.2.1.1	Beijing Asian Modern 3.2 (Pacific RD150)	15	3.6	7	5.1	0.4
L2.2.1	**Beijing Asian Modern 4**	32	7.6	8	5.8	0.5
	other beijing non-classified	40				-
L4.3.3	LAM RD115			1	0.7	-
L4.4.2				5	3.6	-
L4.5	**RD122**	40	9.5	5	3.6	**0.035**
L4.2.2		14	3.3			-
L3	Central Asian (CAS)	6	1.4			-
Total number of isolates		420		137		

* Genotypes detected in >7% in at least one of the populations are in bold. See Materials and Methods, Section 4.4. below, for phylogenetic markers of genotypes and subtypes. ** Significant *p* values (<0.05) are in bold.

**Table 8:** Comparison of pediatric versus adult *M. tuberculosis* populations from Russia (see also Figure S3).

Genotype/Subtypes *	Russia Adult [43], *Number*	Russia Adult [43], %	Russia Children, This Study, *Number*	Russia Children, %	*p*
Beijing Asian Ancestral 1	3	2.9	0	0	-
Beijing Asian Ancestral 2	5	4.8	1	1.7	0.3
Beijing Asian Ancestral 3	0	0	1	1.7	-
Beijing Asian Modern other	3	2.9	1	1.7	0.6
**Beijing B0/W148**	30	28.6	16	26.7	0.8
**Beijing Central Asian/Russian**	39	37.1	31	51.7	0.07
Haarlem	1	1.0	2	3.3	0.3
**Ural**	7	6.7	1	1.7	0.2
LAM	4	3.8	1	1.7	0.4
S	1	1.0	2	3.3	0.3
**L4 other**	12	11.4	4	6.7	0.3
Total number of isolates	105		60		

* See Materials and Methods, Section 4.4. below, for phylogenetic markers of genotypes and subtypes. Groups at >6% are shown in bold.

## Data Availability

All data of this study are presented in this article and its Supplementary material. The WGS Data are available in the Short Reads Archive of NCBI (project numbers PRJNA786957 and PRJNA940524).

## Supplementary Materials

The supporting information can be downloaded at: https://www.mdpi.com/article/10.3390/ijms241210302/s1.
