# Supplementary material for: Insight into Population Structure and Drug Resistance of Pediatric Tuberculosis Strains from China and Russia Gained through Whole-Genome Sequencing
Source: Int J Mol Sci. 2023 Jun 18;24(12):10302. doi: 10.3390/ijms241210302 (PMC10299545; doi:10.3390/ijms241210302)
Supplement: Supplementary file 1 [file ijms-24-10302-s001.zip › Expanded legend to Figure S1.pdf]

Expanded Legend to Figure S1

Figure S1. ML dendrogram (with bootstrap values) of Russian and Chinese pediatric *M. tuberculosis* strains.

The bootstrap values are shown if > 80 %. The 197 sequenced genomes were used along with 40 previously published genomes of lineage 2.2.1 [14] (used as reference to assign our strains to recently described Beijing sublineages in China). The Euro-American lineage forms a group of internal unresolved branches. L2.2.2 Beijing Ancestral 1 is the most distant branch in L2. Most of Beijing Ancestral 2 and Beijing Ancestral 3 constituted a common group of unresolved phylogenetic branches. The Modern Beijing 2.2.1 group has unresolved root branching and represent a star-like evolution, probably due to their relatively recent emergence. The Russian Beijing B0/W148 and Beijing Central Asian strains are included in the large Beijing Asian Modern 2 sublineage and form independent branches.
