# Supplementary material for: Insight into Population Structure and Drug Resistance of Pediatric Tuberculosis Strains from China and Russia Gained through Whole-Genome Sequencing
Source: Int J Mol Sci. 2023 Jun 18;24(12):10302. doi: 10.3390/ijms241210302 (PMC10299545; doi:10.3390/ijms241210302)
Supplement: Supplementary file 1 [file ijms-24-10302-s001.zip › Figure S2.pdf]

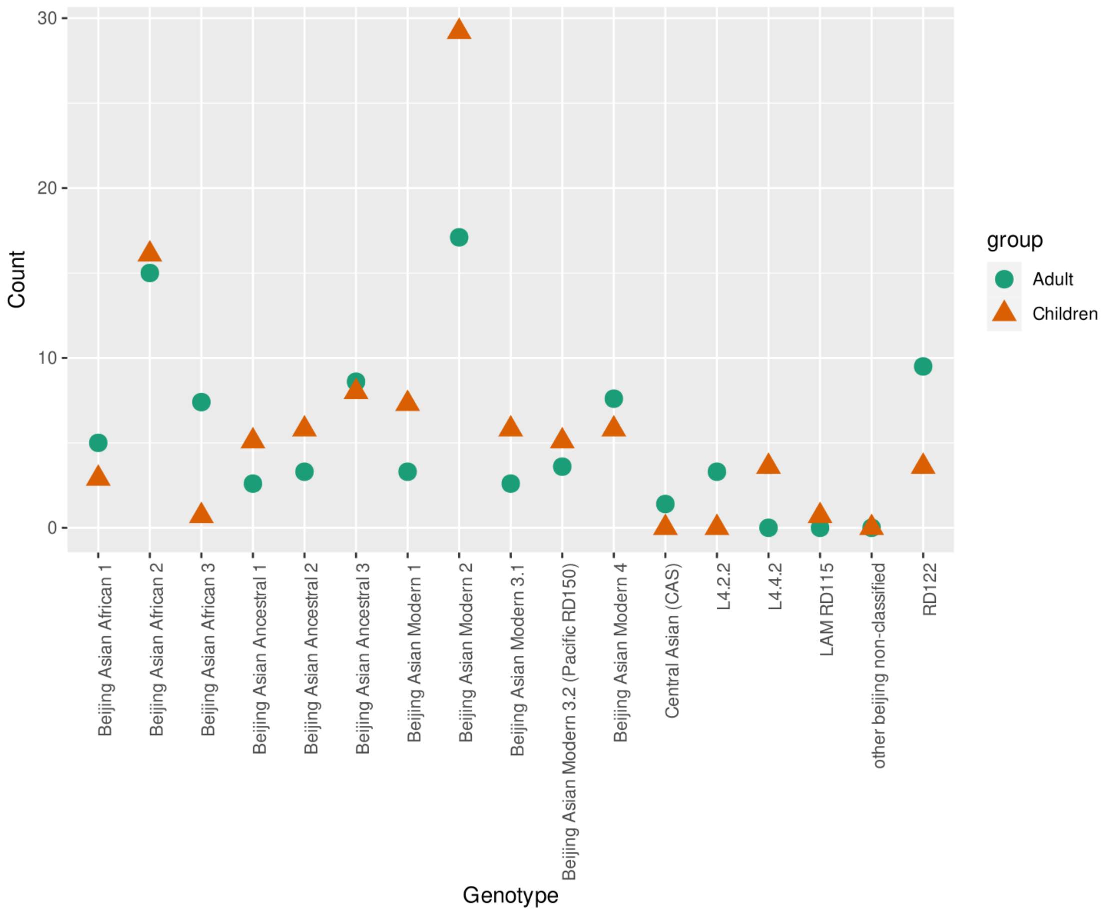


Figure S2: Comparison of pediatric versus adult *M. tuberculosis* populations from China based on data shown in Table 7.
